# Rheumatic Heart Disease-Attributable Mortality at Ages 5–69 Years in Fiji: A Five-Year, National, Population-Based Record-Linkage Cohort Study

**DOI:** 10.1371/journal.pntd.0004033

**Published:** 2015-09-15

**Authors:** Tom Parks, Joseph Kado, Anne E. Miller, Brenton Ward, Rachel Heenan, Samantha M. Colquhoun, Till W. Bärnighausen, Mariana Mirabel, David E. Bloom, Robin L. Bailey, Isimeli N. Tukana, Andrew C. Steer

**Affiliations:** 1 University of Oxford, Oxford, United Kingdom; 2 London School of Hygiene and Tropical Medicine, London, United Kingdom; 3 Fiji Islands Ministry of Health, Suva, Fiji; 4 Townsville General Hospital, Douglas, Australia; 5 Murdoch Children’s Research Institute, Melbourne, Australia; 6 Royal Children’s Hospital, Melbourne, Australia; 7 Centre for International Child Health, University of Melbourne, Melbourne, Australia; 8 Harvard School of Public Health, Boston, Massachusetts, United States of America; 9 Institut National de la Santé et de la Recherche Médicale, Paris, France; University of California San Diego School of Medicine, UNITED STATES

## Abstract

**Background:**

Rheumatic heart disease (RHD) is considered a major public health problem in developing countries, although scarce data are available to substantiate this. Here we quantify mortality from RHD in Fiji during 2008–2012 in people aged 5–69 years.

**Methods and Findings:**

Using 1,773,999 records derived from multiple sources of routine clinical and administrative data, we used probabilistic record-linkage to define a cohort of 2,619 persons diagnosed with RHD, observed for all-cause mortality over 11,538 person-years. Using relative survival methods, we estimated there were 378 RHD-attributable deaths, almost half of which occurred before age 40 years. Using census data as the denominator, we calculated there were 9.9 deaths (95% CI 9.8–10.0) and 331 years of life-lost (YLL, 95% CI 330.4–331.5) due to RHD per 100,000 person-years, standardised to the portion of the WHO World Standard Population aged 0–69 years. Valuing life using Fiji’s per-capita gross domestic product, we estimated these deaths cost United States Dollar $6,077,431 annually. Compared to vital registration data for 2011–2012, we calculated there were 1.6-times more RHD-attributable deaths than the number reported, and found our estimate of RHD mortality exceeded all but the five leading reported causes of premature death, based on collapsed underlying cause-of-death diagnoses.

**Conclusions:**

Rheumatic heart disease is a leading cause of premature death as well as an important economic burden in this setting. Age-standardised death rates are more than twice those reported in current global estimates. Linkage of routine data provides an efficient tool to better define the epidemiology of neglected diseases.

## Author Summary

Rheumatic heart disease is the result of an abnormal immune response to the bacteria *Streptococcus pyogenes*. The disease causes permanent scarring of the heart values, which results in heart failure, stroke and early death. It primarily affects the world’s poorest and most disadvantaged populations and despite the availability of cheap and effective prevention strategies receives little attention from policy-makers and funders. One of the major difficulties has been measuring how many people die prematurely from this disease. Simply counting up deaths is highly inaccurate and so an alternate strategy was needed. Focusing on Fiji in the Western Pacific, we pulled together information from several different health databases using a process called record-linkage. We then worked out how much more frequently rheumatic heart disease patients die than you might expect when comparing them to persons of similar age, gender and ethnicity in the general population. From these data we estimate about twice as many patients were dying from the disease than had been previously suggested. Most of these deaths occurred earlier than was thought with substantial knock-on effects for the economy. On balance we think this strategy for measuring mortality is useful and robust, and it will be increasingly possible to employ it elsewhere.

## Introduction

Rheumatic heart disease (RHD) is the chronic consequence of an aberrant immune response to infection by the bacterial pathogen *Streptococcus pyogenes* that results in permanent scarring of the heart valves. [1] This process, which may manifest clinically as heart failure, stroke and early death, [2] remains a major public health problem in developing countries. [3–5] Despite this, efforts to measure the disease burden and institute control strategies are impeded by the lack of up-to-date epidemiologic data from endemic areas. [4, 6, 7]

Although current global estimates assert there are approximately 275,000 deaths due to RHD each year, [8] deriving such figures has been problematic. [4, 7] In 2005, a WHO report found mortality estimates based on either vital registration data or verbal autopsy techniques to be unreliable, largely because of the difficulty distinguishing RHD-attributable death from other causes of cardiac death. [9] To remedy this, the authors extrapolated from estimates of prevalence and studies of natural history. However, with few current data [10], the only available data were those from urban populations living in the UK, USA and Japan in the early to mid-twentieth century and socially disadvantaged indigenous populations living in Australia and New Zealand today. [9] We therefore sought to measure RHD-attributable mortality in Fiji, a developing nation in the Western Pacific, where a high prevalence of RHD has consistently been reported. [11, 12]

## Methods

### Design

**Fig 1:**
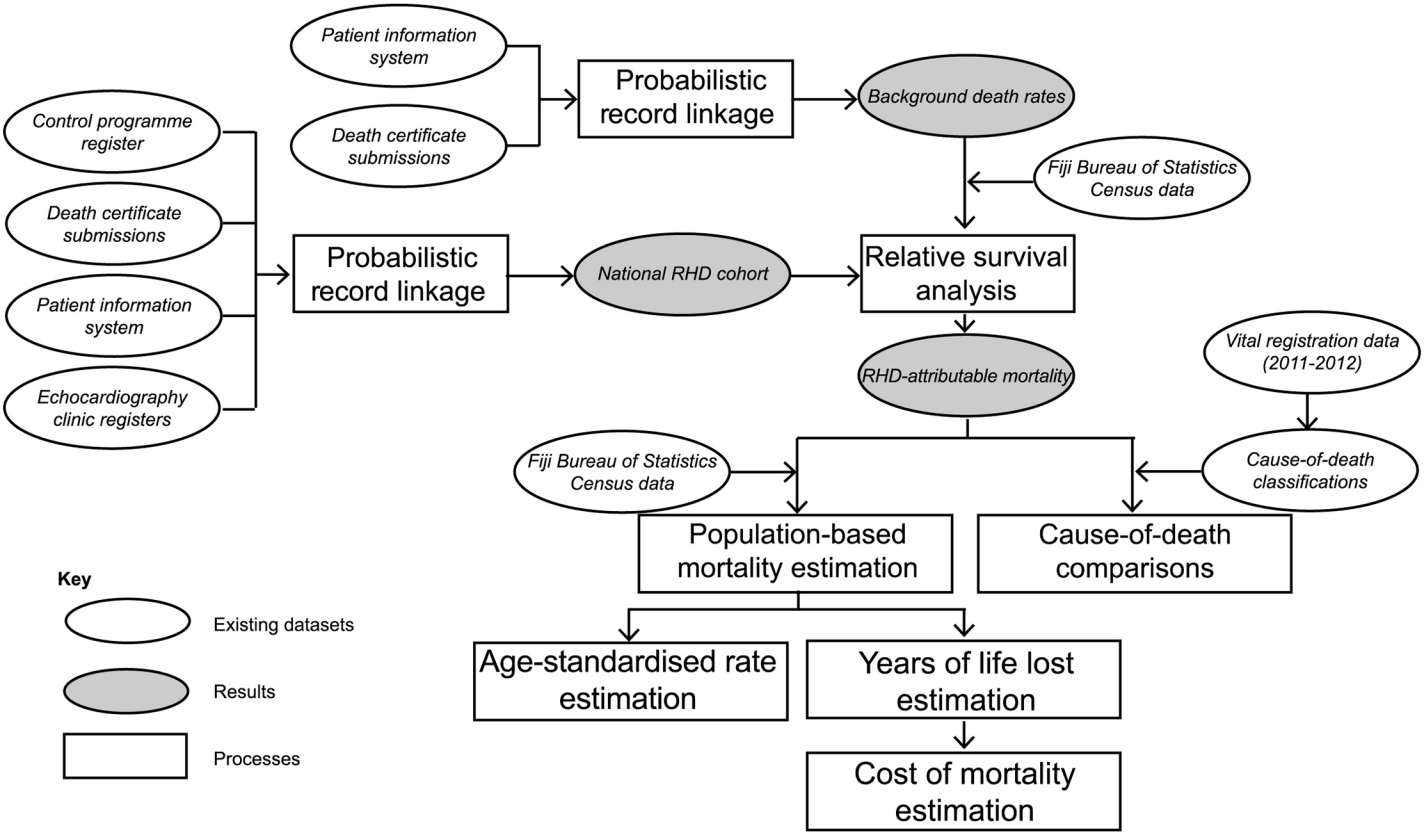
Overall design of the study.

We established a new national and historical cohort of RHD patients in Fiji by probabilistic record-linkage, using diagnostic information and outcome events ascertained from routine clinical and administrative records for the period 2008–2012 (Fig 1). We used relative survival methods to estimate and examine RHD-attributable deaths in persons aged 5–69 years. [13] We then used census data to calculate crude, age-specific and age-standardised RHD-attributable death rates for the wider population, as well as years of life lost (YLL) from which we estimate the cost to the economy. Finally, we compared our RHD mortality estimate with vital registration data for 2011–2012.

### Setting

Fiji is an independent nation located in the Western Pacific with an estimated population of 837,271 at the most recent census in 2007. [14] The population consists of two major ethnic groups, Indigenous iTaukei Fijians (57%) and Fijians of Indian Descent (38%). Fiji is ranked 96th of 186 nations for the composite human development index. [15]

### Sources of data

The routine data used in this study was obtained from four sources: an electronic patient information system, a database of death certificates, a disease control register, and echocardiography clinic registers.

#### Patient information system

Fiji’s Ministry of Health uses a national hospital patient information system (“PATIS”) based on a purpose-built Structure Query Language database. [16] The database contains identifiers and basic demographic information, indexed by a nine-digit national health number (NHN), for the majority of individuals who have sought healthcare at hospital facilities since its inception in 2001. There were 1,133,981 individuals registered on the system to the end of 2012, with 43,435 deaths and 254,503 hospital admissions in the period 2008–2012.

#### Death certificate database

Fiji’s Ministry of Health receives reports of more than 90% of deaths because submission of a medical certificate is mandatory before burial or cremation. [17, 18] There were a total of 30,257 deaths in people aged over four years for the period 2008–2012.

#### Control Programme register

The National RHD Control Programme maintains a centralised register of cases of ARF and RHD, consistent with recommendations by the World Heart Federation, [4] which includes 2,034 individuals registered up to 31st December 2012, of which 984 (48%) registered before 1st January 2008.

#### Echocardiography clinic registers

Additional information was obtained from clinic registers used by the adult and paediatric echocardiography services at the Colonial War Memorial Divisional Hospital in Suva and Lautoka Divisional Hospital in Lautoka. With the vast majority of echocardiography performed at these two centres, and the vast majority of procedures reportedly documented in these registers, it was anticipated that this resource would capture nearly all new echocardiographic diagnoses in the country. These handwritten registers include, at a minimum, the patient’s name, the date of the echocardiogram and a summary of the findings and most entries included the NHN and usually either the date of birth or at least the birth year or the age. For the period 2008–2012 there were 2,126 echocardiograms potentially relevant to RHD relating to 1,440 individuals before duplicates had been removed. The echocardiographer’s interpretation was accepted, provided that nothing to the contrary was documented, with echocardiograms classified as: 1) Firm evidence of RHD; 2) Other evidence of RHD; and 3) Evidence of prior cardiac surgery (S1 Table).

### Record-linkage

Information that referred to the same person was amalgamated from the four datasets using identifier fields. [19] We designed and calibrated a probabilistic record-linkage procedure using 1,406 known duplications in the patient information system from which we calculated the sensitivity and specificity (S1 Box). In its final configuration, our procedure identified the known duplications with sensitivity of 91.4% and specificity of 99.9% with record pairs considered a match if they achieved a posterior probability of over 50%. Stata® code for the procedure is available for download at: http://users.ox.ac.uk/~clme1250/data_linkage/linkage.html.

Our first step was to find at least one match in the patient information system for each record available from the death certificate database, the control programme register and the echocardiography clinic registers. In the absence of a universal identifier, we used a variety of identifier fields in the records including names, dates and demographics to detect pairs of records that referred to the same person (S2 Table). After cleaning and standardisation of names, dates and other identifier fields, we performed an initial shortlisting step (termed ‘blocking’) by finding groups of individuals of similar age with similar names. Next we compared identifiers within each pair or shortlisted records and classified them as being in agreement, partial agreement, disagreement or missing. [20] These classifications allowed for out-of-order names and dates as well as abbreviations and spelling discrepancies of names. [21] We then undertook a further blocking step using combinations of the identifier classifications to define smaller blocks that were expected to contain 5–75% true matches based on the number of pairs per search record. Next, for each block, we estimated the likelihood that each combination or pattern of classifications indicated a true match under the Fellegi-Sunder model of record-linkage; [22] match and nonmatch probabilities were estimated using an expectation maximisation algorithm as previously described. [23] Finally, we obtained a posterior probability of a match by multiplying the raw likelihood by an estimate of prior probability obtained from the product of: 1) The probability that a random pair represented a match, which equated to the reciprocal of the size of the final block in which that pair was found [20]; 2) An estimate of the probability that a given individual had actually been registered in the patient information system [20], based on their locality of residence, age, gender and ethnicity; and 3) For pairings with death certificates only, the probability an individual had died that year based on their age, gender and ethnicity. [24] Record pairs achieving a posterior probability of 50% or more in at least one block were consider a match.

Once links had been identified, we repeated the procedure to confirm or refute the merger of two or more records in the patient information system. Because it was impractical to search for duplicates across the entire patient information system, we limited this search for duplicates to a shortlist of records that were potentially relevant because of a possible match or because they contained useful clinical information such as a relevant admission diagnosis. Finally we pulled clinical information from each of the sources into a single linked record and deleted identifiers. If there was a discrepancy between the records, the patient information system was assumed to be correct unless that field was missing. Where there were discrepancies amongst two or more patient information system records these fields were set to missing.

### Cohort and outcomes

We focused on 2008–2012 because the most complete data were available for this period. The study was restricted to people aged 5–69 years because RHD is expected to cause very few deaths before five years of age and cause-of-death information can be unreliable in old age. Based on diagnostic information in control programme records, echocardiographic data, hospital discharges and death certificates, any individual with follow-up at ages 5–69 years who had a least one record of a diagnosis of either RHD or its precursor acute rheumatic fever (ARF) was eligible for inclusion (S2 Box). We assumed the onset of disease was in childhood irrespective of the date the individual became known to clinical services, circumventing potential bias due to late presentation. The primary outcome was the time to all-cause death defined by a date of death in either the patient information system or the death certificate database. The secondary outcomes were cause-specific death defined: 1) Narrowly, where the underlying cause-of-death was ascribed to an International Statistical Classification of Diseases and Related Health Problems 10th Revision (ICD10) code pertaining to RHD or ARF; and 2) Broadly, where the death certificate listed an ICD10 code pertaining to either RHD, an alternative description of valvular heart disease, or a cardiac complication thereof (i.e. heart failure, stroke, infective endocarditis, arrhythmia) as an immediate or underlying cause-of-death, without listing ischaemic heart disease. We surmised there was no loss to follow-up because the linked patient information system and the death certificate database would include most deaths in the country during 2008–2012. Deaths in the cohort that went undetected due to, for example, emigration, would have led us to under-estimate RHD-attributable mortality.

### Statistical methods

Data were inspected for missing and outlying points, categorical variables were tabulated and continuous variables were summarised in histograms. The cohort data were expanded into thirteen five-year age categories and standardised mortality ratios (SMR) and relative survival rates were calculated by applying the background age category, gender and ethnicity specific death rates to the cohort. [13] Background death rates were calculated using the count of deaths in the patient information system and death certificates after duplicates had been removed divided by estimates of population using data from the Fiji Bureau of Statistics. [14] Before application these rates were checked for adherence with expectations by performing of standard quality control checks such as log rate versus age category. We assumed time-to-death from RHD was independent of time-to-death from other causes, consistent with the previous application of relative survival to RHD mortality, [25] but as an alternative performed competing risk analyses based on broad and narrow definitions for cause-specific death. Using Poisson regression, we modelled the relative risk conferred by decade of age, gender and ethnicity, adjusting for calendar year of exit to account for artifactual differences in the background death rates during the five year study.

We used excess mortality in the cohort to investigate RHD-attributable mortality before age 70 years in the wider population under the assumptions that all RHD patients in the country had been detected and no RHD deaths occurred before age 5 years. We calculated crude, age-specific and age-standardised RHD-attributable death rates for the general population, deriving 95% confidence intervals (CI) using Poisson, and using estimates of population from the Fiji Bureau of Statistics as the denominator. We tested the robustness of these results by changing the stringency of the record-linkage procedure through adjusting the posterior probability at which record pairs were considered to match. We also calculated YLL in each of the thirteen age categories using WHO Life Tables [26] and calculated rates for the wider population. We used the WHO World Standard population for direct standardisation. [27] Finally, the availability of ICD10 coded underlying cause-of-death classifications during 2011–2012 permitted comparison of our estimates with the number of reported deaths due to RHD itself and other conditions. To make the comparison, we collapsed ICD10 codes into the diagnostic categories used in the Global Burden of Disease (GBD) project, [28] grouping ill-defined codes separately. [29] To maximise parity we recalculated the number of RHD-attributable deaths based only on deaths associated with a death certificate.

### Estimates of costs

We used a human capital approach [30] to define the cost of premature mortality to Fiji. We estimate wider cost of illness due to RHD/ARF in Fiji in a separate paper, providing further details and justification for the methods. [31] We estimated the cost of a death in each of the five-year age categories by multiplying life-expectancy in years using Fiji’s per-capita gross domestic product for the year in which the death occurred as estimated by the World Bank [32] discounting 3% each year. For each category and year of the study, we then multiplied this estimate by the excess deaths and summed the results to obtain the total cost of RHD-attributable deaths over the five-year period.

### Systematic review

We searched for population-based studies of RHD mortality published in a thirty year period, 1985–2014, in Ovid Medline, Embase and Global Health (S4 Fig). We used the search terms “rheumatic heart disease” and “mortality” with a previously described filter to detect studies from developing countries. [33] We excluded case reports, case series, studies focused on valve surgery and studies not specific to RHD.

### Research ethics

Permission for the study was granted by the Fiji National Research Ethics Review Committee (2013–89) in addition to the Oxford Tropical Research Ethics Review Committee (1055–13). Once the record-linkage procedures were complete, all data analysed were anonymised.

## Results

**Fig 2:**
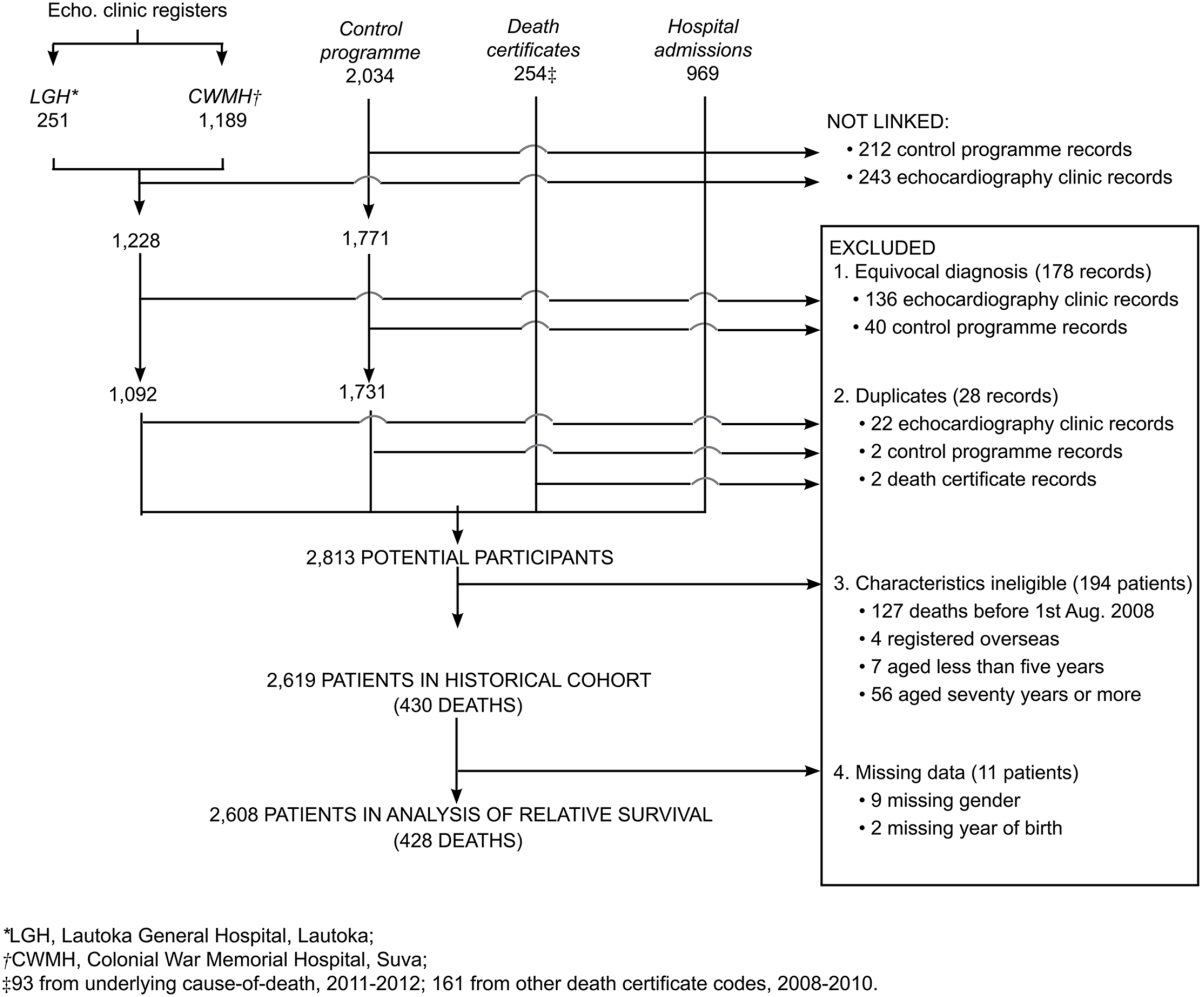
Flow diagram of ascertainment.

In total, 1,773,999 records were available, including 34,773 records that pertained to a death. Links were identified in the patient information system for 87.1% of control programme records, 85.3% of echocardiography clinic records and 66.0% of death certificates (S3 Table). After selecting eligible individuals with an RHD or ARF diagnosis, a cohort of 2,619 individuals remained for analysis (Fig 2). Of these, 1,038 (39.6%) were present in more than one database (S1 Fig). Characteristics are summarised in Table 1; the person-time observed totalled 11,537.5 person-years.

**Table 1:** Characteristics of the cohort by survival.

	**Survived**	**Died**	**p-value**
	n = 2189	n = 430	
Age at entry, median (IQR*)	16.4 (9.6–32.2)	41.5 (26.2–53.6)	<0.001†
Gender, n (%)			0.11‡
*Male*	944 (43.3)	204 (47.4)	
*Female*	1236 (56.7)	226 (52.6)	
Ethnicity, n (%)			0.008‡
*(Indigenous) iTaukei*	1404 (64.4)	289 (67.4)	
*Indian descent*	629 (28.9)	128 (29.8)	
*Other*	146 (6.7)	12 (2.8)	
Known to control programme, n (%)	1460 (66.7)	137 (31.9)	<0.001‡
Echocardiographic data, n (%)			<0.001‡
*Attendance*	392 (17.9)	32 (7.4)	
*Mitral stenosis*	333 (15.2)	77 (17.9)	
*Other firm diagnosis§*	196 (9.0)	34 (7.9)	
Cardiac hospitalization, n (%)∣∣	917 (41.9)	312 (72.6)	< 0.001‡
Cardiac surgery, n (%)¶	283 (12.9)	46 (10.7)	0.2‡

* Interquartile range;

† Wilcoxon rank-sum;

‡ *χ* ^2^ test;

§ Refers to any of World Heart Federation criteria definite [41], moderate or severe mitral regurgitation (MR), or mild MR with two or more morphological features at mitral valve;

∣∣ During follow-up;

¶ Before or during follow.

During follow-up, 430 of the 2,619 (16.4%) RHD/ARF patients were linked to a death in the patient information system, the death certificate database or both. This equated to 2.1% of the 20,796 deaths in the general population in the same age bracket during this time. The all-cause unadjusted death rate amongst RHD/ARF patients was 3.7% per year (95% CI 3.4–4.1%). Death rates based on cause-of-death information are summarised in S6 Table. From late childhood onwards, the death rate observed in the cohort exceeded that in the wider population (SMR 8.3, 95% CI 7.5–9.0, S2 Fig). The relative survival was 96.9% (95% CI 96.1–97.5%) at one year and 81.2% (95% CI 79.2–83.0%) at five years (S3 Fig). The risk of death among RHD/ARF patients increased with age over and above background rates; there was also increased risk for both male and iTaukei patients (S4 Table).

**Fig 3:**
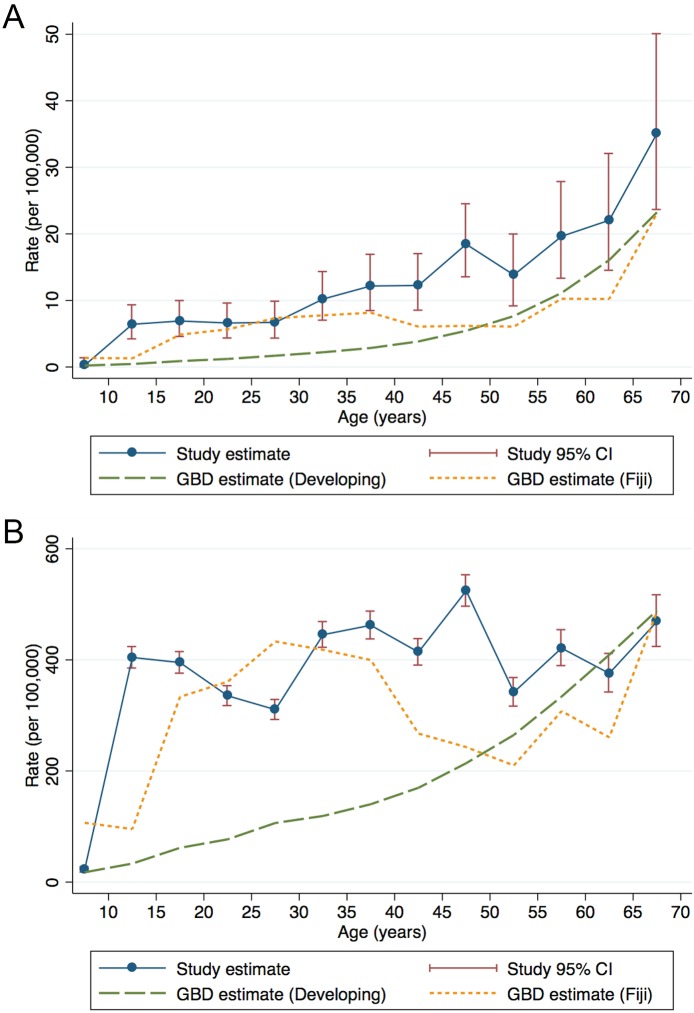
Estimated RHD-attributable death rates at ages 5–69 in Fiji, 2008–2012. A: Death rate, B: YLL rate. Both are plotted at mid-point of age group. Global Burden of Disease (GBD) project estimates for 2010 for all developing countries and for Fiji are plotted for comparison.

Based on the 378 excess deaths, of which 177 (46.8%) occurred before age 40 years, we estimated there were 9.1 RHD-attributable deaths (95% CI 8.2–10.1) per 100,000 person-years in those aged 0–69 years. This estimate remained stable to adjustments in the record-linkage threshold and between the 2008–2010 and 2011–2012 components of the dataset (S7 Table). Age-specific rates of RHD-attributable death increased throughout life (Fig 3A). Standardised to the portion of the WHO World Standard Population aged 0–69 years, our primary estimate equates to 9.9 deaths (95% CI 9.8–10.0) per 100,000 person-years (S5 Table). Additionally, we estimated 323.3 YLL (95% CI 317.9–328.6) per 100,000 person-years, equating to a WHO standardised rate of 331.0 YLL (95% CI 330.4–331.5) per 100,000 person-years (S5 Table). Age-specific RHD-attributable YLL rates were elevated from late childhood onwards (Fig 3B). The cost of these deaths for the five-year period was current Fiji Dollar $58,810,903, which at mid-market rates equates to United States Dollar $30,387,153.

**Fig 4:**
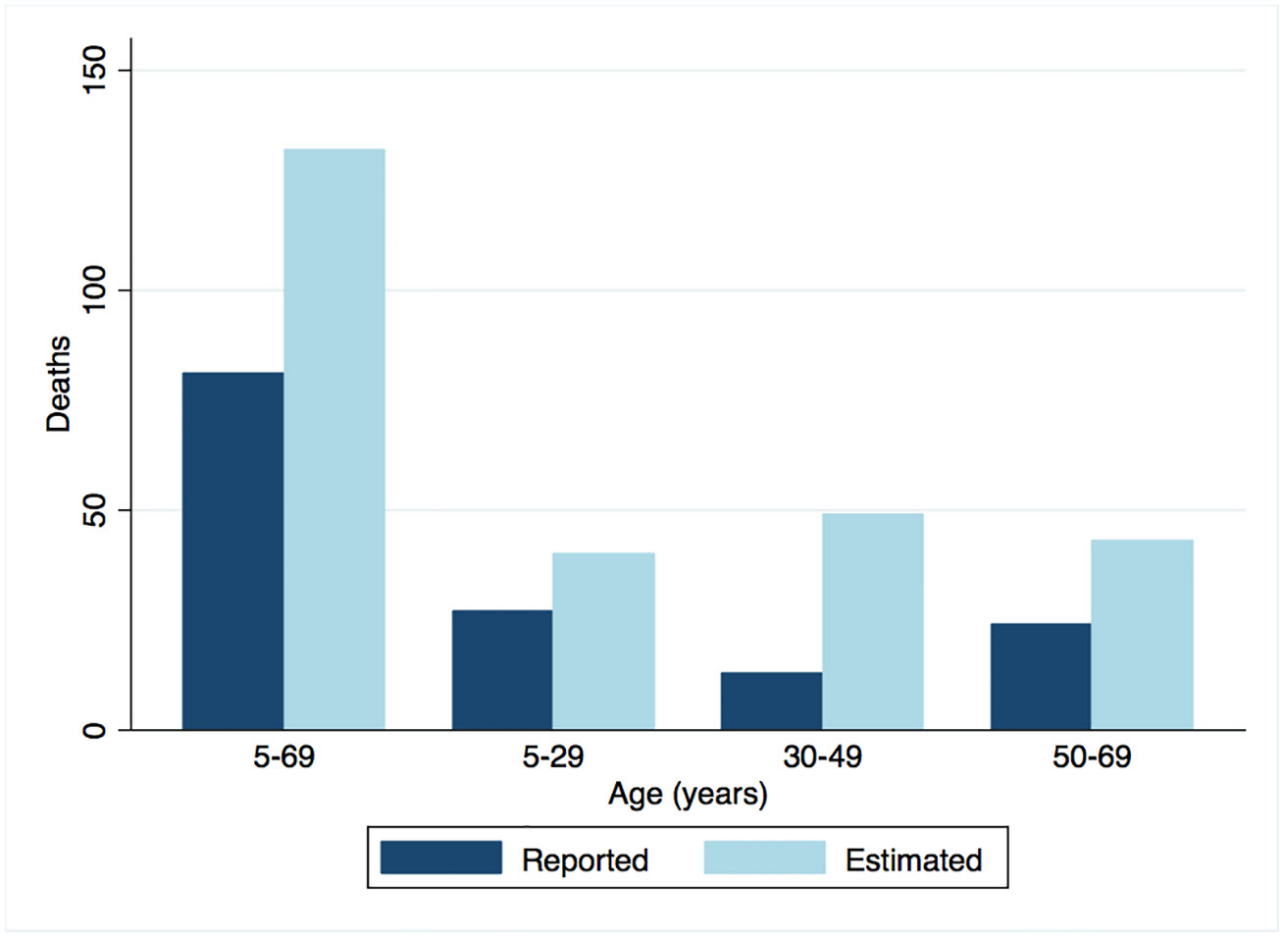
Reported and estimated RHD-attributable deaths by age in Fiji, 2011–2012. Reported counts are derived from ICD10 coding of underlying cause-of-death classifications in vital registration data. Estimated counts are based on excess deaths in the cohort.

Finally, we estimated there were 132 RHD-attributable deaths during 2011–2012 based only on deaths associated with a death certificate, compared to 81 RHD deaths reported in vital registration data (Fig 4). Only five other conditions caused more than 132 deaths in the wider population while ten caused more than 81 deaths (S8 Table). Moreover, only drowning caused more than the 40 deaths attributable to RHD at ages 5–29 years, which was greater than the number attributed to suicide and road injury, both well-recognised causes of death in young people.

## Discussion

These are the first national population-based age-standardised estimates of mortality due to RHD in a developing country (S4 Fig), and confirm that RHD is an important cause of premature death in Fiji leading to a substantial loss of life and economic productivity. The study was made possible by applying record-linkage techniques to the routine clinical and administrative data that are increasingly available in an electronic format in many developing countries. The results are robust to changes in record-linkage thresholds and remain broadly similar throughout the five years the study covers, despite changes in local death reporting practices during this time.

Using the background death rates in the general population, we were able to estimate RHD-attributable mortality by measuring excess mortality. These methods, which are widely used for population-based cancer survival analyses [13], are highly applicable to RHD, a disease for which cause-of-death information is often absent or unreliable. [6, 25] For example, if our results are compared to underlying cause-of-death classifications in vital registration data for 2011–2012, we find 1.6-fold more RHD-attributable deaths at ages 5–69 years, a discrepancy that peaked at 3.8-fold in the 30–49 years age group (Fig 4). This finding is consistent with a recent study of mortality amongst RHD patients in Western Australia which, by reviewing death certificates and other clinical data, concluded a third of RHD-attributable deaths were ascribed to other underlying cause-of-death diagnoses. [34]

Few existing data are available for comparison. One recently published study presents RHD-attributable death rates based on vital statistics for South Africa for the period 1997–2012 during which time the crude all-age death rate declined from 1.2 to 0.7 per 100,000 person-years. [35] As the authors acknowledge, however, the reliability of estimates based on death certification is questionable [35], particularly given that in 1999 both the UK and Japan reported RHD death rates over two-fold higher than this. [9] Interestingly, our age-standardised and age-specific rates are more comparable to those made for the Coloured population in South Africa for 1978–1982, the authors of that report estimating an age-standardised rate of 3.5 and 4.2 per 100,000 for men and women respectively. [36] Moreover, our age-standardised estimate is similar to that reported for Indigenous populations in New Zealand [37] and Alaska [38] during the 1970–1980s although it exceeds death rates reported from New Zealand [39] and Australia [25] more recently. Alternatively, we can compare our results to the estimates made by the GBD project (Fig 3). [28] Directly standardised to the population aged 0–69 years, we report higher death and YLL rates than GBD (S6 Table), the latter amounting to a 2.6-fold difference in the death rate and 2.4 in the YLL rate compared to the GBD’s developing countries estimate. Thus our data not only have important implications for the Pacific region but also, if generalisable to other developing countries, for global summary estimates.

Although the results appear reasonable, they have some limitations. First, there are potential shortcomings to using background death rates to estimate exposure-attributable mortality. If we were wrong to assume time-to-death from RHD was independent of time-to-death from other causes, we may have over-estimated RHD mortality; however, the impact would be small and alternatives such as cause-specific survival remain unsatisfactory. [13] Second, the cohort was heterogeneous with respect to the chronicity and severity of the illness, and there was no means to distinguish new onset from relapses of chronic disease. This led us to make the conservative assumption that participants were at risk from childhood onwards, which would lead to an under-estimate of mortality if the true onset was later. Third, the study was retrospective and relied on routine clinical and administrative data, which are likely to have contained errors. In particular, while death certificate submission in Fiji is relatively complete [17, 18], data pertaining to underlying cause-of-death should be interpreted with some caution; [18] our comparison with other causes of death may slightly exaggerate the disease’s importance. Fourth, these data provide neither sufficient detail nor follow-up to answer important outstanding questions about why such a burden of disease exists in this setting. For example, the discrepancy between the two largest ethnic groups remains unexplained, although a number of cultural, socioeconomic, geographic and potentially biological factors may contribute. Finally, we are unable to report on RHD-attributable deaths beyond age 69 years although we note GBD estimated a fifth of global RHD deaths occurred in this age group.

By illustrating the high burden of premature death due to RHD in Fiji, these data help substantiate the assertion that RHD remains, on a global scale, [6] an important cause of mortality. By using record-linkage techniques, we have demonstrated that routine clinical and administrative data can be used to quantify the impact of RHD in developing countries, a finding which has important implications for both research and disease control.

## Supporting Information

S1 Table Echocardiography criteria for review of clinic registers. (PDF)

S2 Table Identifiers used to link records to and remove duplicates from the patient information system. (PDF)

S3 Table Outcome of record-linkage procedures. (PDF)

S4 Table Multivariate Poisson relative survival model. (PDF)

S5 Table Deaths and years of life lost due to RHD with denominator and standard population by age in Fiji, 2008–2012. (PDF)

S6 Table Rates of death due to RHD based on relative survival and cause-of-death by narrow and broad definitions in Fiji, 2008–2012. (PDF)

S7 Table Crude death rates due to RHD in the general population with sensitivity analyses in Fiji, 2008–2012. (PDF)

S8 Table Leading collapsed cause-of-death diagnoses in vital registration data compared to estimated RHD-attributable deaths by age in Fiji, 2011–2012. (PDF)

S1 Fig Venn diagram of overlap between databases. Note that the size of the ellipses is not proportional.
(TIF)

S2 Fig Ratio of observed to expected deaths for RHD/ARF patients by gender and age group, in Fiji, 2008–2012. Plotted at mid-point of age group with 95% CIs.
(TIF)

S3 Fig Cumulative percentage relative and expected all-cause death in the cohort. Plotted using local weighted scatter plot smoothing.
(TIF)

S4 Fig Identification of articles for the systematic review. (TIF)

S1 Box Calibration study for record linkage procedure. (PDF)

S2 Box Inclusion and exclusion criteria. (PDF)

S1 Checklist STROBE Checklist. (PDF)
