# Supplementary material for: Rheumatic Heart Disease-Attributable Mortality at Ages 5–69 Years in Fiji: A Five-Year, National, Population-Based Record-Linkage Cohort Study
Source: PLoS Negl Trop Dis. 2015 Sep 15;9(9):e0004033. doi: 10.1371/journal.pntd.0004033 (PMC4570761; doi:10.1371/journal.pntd.0004033)
Supplement: S1 Table — (PDF) [file pntd.0004033.s001.pdf]

S1 Table. Echocardiography criteria for review of clinic registers.

Group	Subgroup	Applicable ages	Diagnosis stated	Accepted findings
Firm evidence	WHF Definite	$\geq$ 5 years	None*	As per guidelines
	Mitral stenosis	$\geq$ 5 years	None*	Any MS
	Other	5–29 years	None*	Any of:<br>• Mod. or sev. MR<br>• Mod. or sev. AR<br>• Mild MR with $\geq$2 morphological features of the MV
		$\geq$ 30 years	Mandatory	
Other evidence	WHF Borderline	5–20 years	None*	As per guidelines
	Other	$\geq$ 5 years	Mandatory	Any of:<br>• Mild MR<br>• Mild AR<br>• Moderate TR
Evidence of prior surgery	N/A	$\geq$ 5 years	None*	Prosthesis or other information documented

*Providing no contradictions; WHF, World Heart Federation; MR, mitral regurgitation; MV, mitral valve; AR, aortic regurgitation; TR, tricuspid regurgitation; Mod., moderate; Sev., severe.
