# Supplementary material for: Rheumatic Heart Disease-Attributable Mortality at Ages 5–69 Years in Fiji: A Five-Year, National, Population-Based Record-Linkage Cohort Study
Source: PLoS Negl Trop Dis. 2015 Sep 15;9(9):e0004033. doi: 10.1371/journal.pntd.0004033 (PMC4570761; doi:10.1371/journal.pntd.0004033)
Supplement: S2 Table — (PDF) [file pntd.0004033.s002.pdf]

**S2 Table. Identifiers used to link records to and remove duplicates from the patient information system.**

| Identifier field | Disease control programme | Echo. clinic registers | Death certificates 2011–2012* | Patient information system† |
|---|---|---|---|---|
| National health number | X | X | X | |
| Individual's name | X | X | X | X |
| Father's name | X | | X | X |
| Year of birth | X | X | X | X |
| Date of birth | X | X | | X |
| Date of death | | | X | X |
| Gender | X | X | X | X |
| Division of residence | X | X | X | X |
| Subdivision of residence | | | X | X |
| Zone/area of residence | | | | X |

*Due to prior indexing and cross-checking by Ministry of Health staff, links from the patient information system to death certificates issued during 2008–2010 were based on NHN alone. †Identifiers used for removal of duplicates within the patient information system.
