# Supplementary material for: Rheumatic Heart Disease-Attributable Mortality at Ages 5–69 Years in Fiji: A Five-Year, National, Population-Based Record-Linkage Cohort Study
Source: PLoS Negl Trop Dis. 2015 Sep 15;9(9):e0004033. doi: 10.1371/journal.pntd.0004033 (PMC4570761; doi:10.1371/journal.pntd.0004033)
Supplement: S3 Table — (PDF) [file pntd.0004033.s003.pdf]

S3 Table. Outcome of record-linkage procedures.

Database	Records, n	Linked records (%) Study	Tolerant	Stringent	Median *match weight* (IQR*) of links†	Duplicates removed, n (%)
Control programme	2,304	1,771 (87.1)	1,786 (77.5)	1,747 (75.8)	47.6 (37.2–49.7)	2 (0.1)
Echo. clinic register	1,440	1228 (85.3)	1282 (89.0)	1252 (86.9)	56.7 (53.7–79.7)	22 (1.8)
Death certificates (2011–2012)	12,244	8,087 (66.0)	8,237 (67.3)	7,917 (64.7)	34.6 (19.1–45.8)	117 (1.4)

*IQR, interquartile range; †At study threshold, i.e. *match weight* $(W) \geq 0$, probability $(P) \geq 50\%$, where $P = \frac{2^W}{1+2^W}$
