# Supplementary material for: Rheumatic Heart Disease-Attributable Mortality at Ages 5–69 Years in Fiji: A Five-Year, National, Population-Based Record-Linkage Cohort Study
Source: PLoS Negl Trop Dis. 2015 Sep 15;9(9):e0004033. doi: 10.1371/journal.pntd.0004033 (PMC4570761; doi:10.1371/journal.pntd.0004033)
Supplement: S4 Table — (PDF) [file pntd.0004033.s004.pdf]

S4 Table. Multivariate Poisson relative survival model.

	Relative risk∗	95% CI	P-value
Male	1		
Female	0.72	(0.58–0.89)	0.003
iTaukei ethnicity	1		
Indian descent	0.55	(0.43–0.71)	¡ 0.001
Other or missing ethnicity	0.34	(0.17–0.70)	0.003
Aged, 5–9 years	1		
10–19 years	3.1	1.7–5.6	¡ 0.001
20–29 years	6.3	3.4–11.5	¡ 0.001
30–39 years	10.7	5.9–19.4	¡ 0.001
40–49 years	14.4	8.0–26.0	¡ 0.001
50–59 years	16.6	8.9–31.0	¡ 0.001
60–69 years	31.0	16.5–58.3	¡ 0.001
Log likelihood	-273.6		

∗Adjusted for calendar year of exit.
