# Supplementary material for: Rheumatic Heart Disease-Attributable Mortality at Ages 5–69 Years in Fiji: A Five-Year, National, Population-Based Record-Linkage Cohort Study
Source: PLoS Negl Trop Dis. 2015 Sep 15;9(9):e0004033. doi: 10.1371/journal.pntd.0004033 (PMC4570761; doi:10.1371/journal.pntd.0004033)
Supplement: S5 Table — (PDF) [file pntd.0004033.s005.pdf]

S5 Table. Deaths and years of life lost due to RHD with denominator and standard population by age in Fiji, 2008–2012.

Age	Study estimates					GBD project rates				WHO Standard Popn	
(years)	Deaths	Death rate	YLL*	YLL rate	Fiji Popn†	Death (Devel.)‡	YLL (Devel.)‡	Death (Fiji)	YLL (Fiji)	All ages§ (%)	Age 0–69‖ (%)
0-4	0.0	0.0	0.0	0.0	84926	0.4	32.3	0.5	40.5	8.86	9.35
5-9	1.4	0.3	89.8	22.4	80020	0.2	17.6	1.4	106.8	8.69	9.17
10-14	27.4	6.5	1701.2	404.4	84145	0.5	33.4	1.3	95.4	8.60	9.08
15-19	28.4	7.0	1601.0	395.1	81040	0.9	61.7	4.9	333.5	8.47	8.94
20-24	26.7	6.5	1370.1	335.3	81722	1.2	77.0	5.7	360.8	8.22	8.67
25-29	24.5	6.6	1157.9	310.3	74621	1.7	100.4	7.4	433.0	7.93	8.37
30-34	33.5	10.4	1439.4	445.4	64629	2.2	119.0	7.8	418.0	7.61	8.03
35-39	35.0	12.2	1328.7	462.1	57501	2.9	140.2	8.2	399.7	7.15	7.55
40-44	35.0	12.2	1183.1	414.0	57158	3.8	169.7	6.1	267.4	6.59	6.95
45-49	47.5	18.6	1336.2	524.4	50960	5.4	214.0	6.2	243.0	6.04	6.37
50-54	28.4	14.0	692.0	341.8	40493	7.7	264.9	6.1	209.9	5.37	5.67
55-59	31.1	19.7	665.2	421.2	31584	11.1	333.9	10.3	307.8	4.55	4.80
60-64	27.1	22.1	460.0	375.7	24490	16.1	409.2	10.2	260.5	3.72	3.93
65-69	29.9	34.9	400.7	468.6	17102	23.2	488.9	23.1	486.6	2.96	3.12
Age-standardised¶	–	9.9	–	331.0	–	3.8	136.2	5.8	269.5	–	

Rates are given per 100,000 person years. * YLL, Years of life lost; † Estimated midpoint population in Fiji; ‡ GBD estimate for all developing countries; ‖ Fraction of WHO Standard Population aged less than 70 years in age category; ¶Age-standardized in population under age 70 years.
