# Supplementary material for: Rheumatic Heart Disease-Attributable Mortality at Ages 5–69 Years in Fiji: A Five-Year, National, Population-Based Record-Linkage Cohort Study
Source: PLoS Negl Trop Dis. 2015 Sep 15;9(9):e0004033. doi: 10.1371/journal.pntd.0004033 (PMC4570761; doi:10.1371/journal.pntd.0004033)
Supplement: S6 Table — (PDF) [file pntd.0004033.s006.pdf]

S6 Table. Rates of death due to RHD based on relative survival and cause-of-death by narrow and broad definitions in Fiji, 2008–2012.

Outcome	Analysis	Period	Cohort	Observed deaths	Excess deaths	Annual net survival	SMR* (95% CI)	CDR† (95% CI)	Assumption
Record of death in primary sources‡	Relative survival	2008–2012	2619	430	378	96.2%	8.3 (7.5–9.0)	9.1 (8.2–10.1)	Independence
Narrow cause-specific§	Competing risk	2011–2012	2333	99‖	80	98.2%	4.2 (3.4–5.3)	4.8 (3.8–6.0)	Accurate cause-of-death
Broad cause-specific¶	Competing risk	2008–2010	2588	220**	188	97.3%	5.8 (5.1–6.7)	7.5 (6.5–8.7)	Accurate cause-of-death
All-cause death	Crude survival	2008–2012	2619	430	430	96.3%	–	10.4 (9.4–11.4)	All death attributable to RHD

* SMR, Standardised mortality ratio; † CDR, Crude death rate from RHD in the general population per 100,000 person-years before age 70 years; ‡ Death recorded on death certificate and/or patient information system (primary endpoint); § ICD10 code for ARF and RHD as underlying cause-of-death – data only available for 2011–2012; ‖ One death excluded due to missing birth date; ¶ ICD10 code for ARF, RHD or complication as immediate or underlying cause-of-death on death certificate in the absence of an ischemic heart disease code – data only available for 2008–2010; ** One death excluded due to missing birth date.
