# Supplementary material for: Rheumatic Heart Disease-Attributable Mortality at Ages 5–69 Years in Fiji: A Five-Year, National, Population-Based Record-Linkage Cohort Study
Source: PLoS Negl Trop Dis. 2015 Sep 15;9(9):e0004033. doi: 10.1371/journal.pntd.0004033 (PMC4570761; doi:10.1371/journal.pntd.0004033)
Supplement: S7 Table — (PDF) [file pntd.0004033.s007.pdf]

S7 Table. Crude death rates due to RHD in the general population with sensitivity analyses in Fiji, 2008–2012.

Condition	Cohort	Observed deaths	Excess deaths	SMR* (95% CI)	CDR† (95% CI) per 100,000
Primary analysis (≥50% prob.‡)	2619	430	378	8.3 (7.5–9.0)	9.1 (8.2–10.1)
Tolerant linkage (≥10% prob.‡)	2644	445	393	8.6 (7.8–9.4)	9.5 (8.6–10.4)
Stringent linkage (≥90% prob.‡)	2595	418	369	8.0 (7.3–8.8)	8.9 (8.0–9.8)
2008–2010 data	2588	269	215	5.0 (4.4–5.6)	8.6 (7.5–9.9)
2011–2012 data§	2333	155	137	8.4 (7.1–9.8)	8.2 (6.9–9.8)

* SMR, Standardized mortality ratio; † CDR, Crude death rate from RHD in the general population per 100,000 person-years before age 70 years; ‡ Probability threshold for two records to be considered a match used in all stages of the record-linkage process; § Extra-linkage step required because death-certificates not index by national health number.
