# Supplementary material for: Rheumatic Heart Disease-Attributable Mortality at Ages 5–69 Years in Fiji: A Five-Year, National, Population-Based Record-Linkage Cohort Study
Source: PLoS Negl Trop Dis. 2015 Sep 15;9(9):e0004033. doi: 10.1371/journal.pntd.0004033 (PMC4570761; doi:10.1371/journal.pntd.0004033)
Supplement: S8 Table — (PDF) [file pntd.0004033.s008.pdf]

S8 Table. Leading collapsed cause-of-death diagnoses in vital registration data compared to estimated RHD-attributable deaths by age in Fiji, 2011–2012.

Age (years)	Rank	Cause-of-death*	Deaths§
5–69	1	Diabetes mellitus	1979
	2	Ischemic heart disease	1035
	3	Breast cancer	181
	4	Asthma	158
	5	Cerebrovascular disease	147
	6	Cervical cancer	131
	7	Road injury	94
	8	Drowning	93
	9	Liver cancer	90
	10	Leptospirosis	86
	11	*RHD-reported*†	81
	-	*RHD-attributable*‡	132
5–29	1	Drowning	52
	2	Road injuries	30
	3	Suicide	28
	4	*RHD-reported*†	27
	5	Leptospirosis	26
	-	*RHD-attributable*‡	40
30–49	1	Diabetes mellitus	301
	2	Ischemic heart disease	277
	3	Breast cancer	69
	4	Cervical cancer	57
	5	Road injuries	43
	8	*RHD-reported*†	13
	-	*RHD-attributable*‡	49
50–69	1	Diabetes mellitus	1,662
	2	Ischemic heart disease	742
	3	Breast cancer	111
	4	Asthma	107
	5	Cerebrovascular disease	105
	6	Cervical cancer	71
	7	Liver cancer	67
	8	Diarrheal diseases	45
	9	Chronic obstructive pulmonary disease	40
	10	Lung cancer	38
	20	*RHD-reported*†	24
	-	*RHD-attributable*‡	43

* Leading ten causes for ages 5–69 years and 50–69 years and leading five causes otherwise; † "RHD-reported", deaths reported as RHD/ARF in vital registration data. ‡ "RHD-attributable", estimate of RHD/ARF in this study; § 2,173 of 8,161 (26.6%) deaths with ill-defined codes are excluded.
