# Supplementary figures and images for: Rheumatic Heart Disease-Attributable Mortality at Ages 5–69 Years in Fiji: A Five-Year, National, Population-Based Record-Linkage Cohort Study
Source: PLoS Negl Trop Dis. 2015 Sep 15;9(9):e0004033. doi: 10.1371/journal.pntd.0004033 (PMC4570761; doi:10.1371/journal.pntd.0004033)

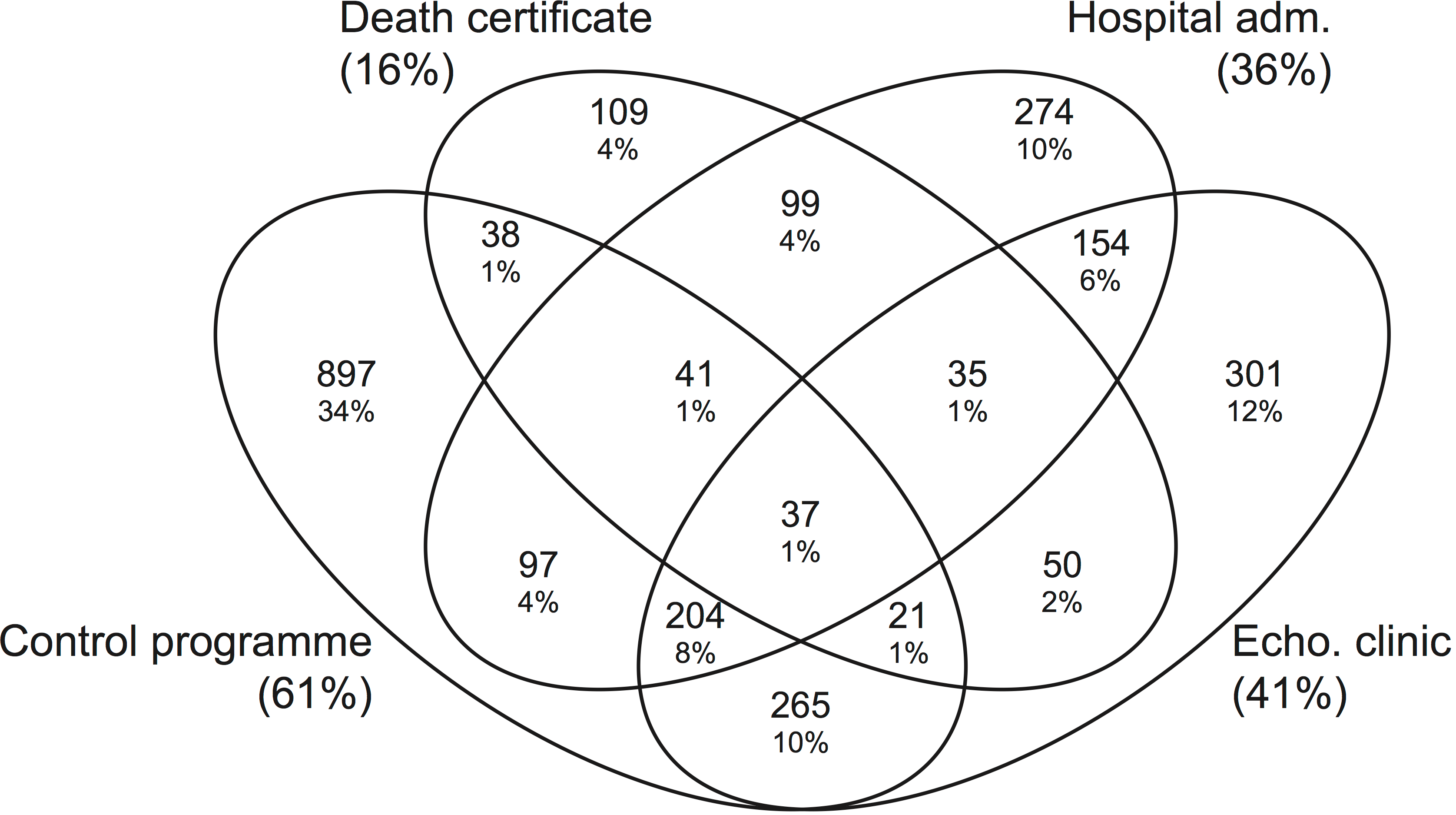

Supplement: S1 Fig — Note that the size of the ellipses is not proportional. (TIF) [file pntd.0004033.s009.tif]

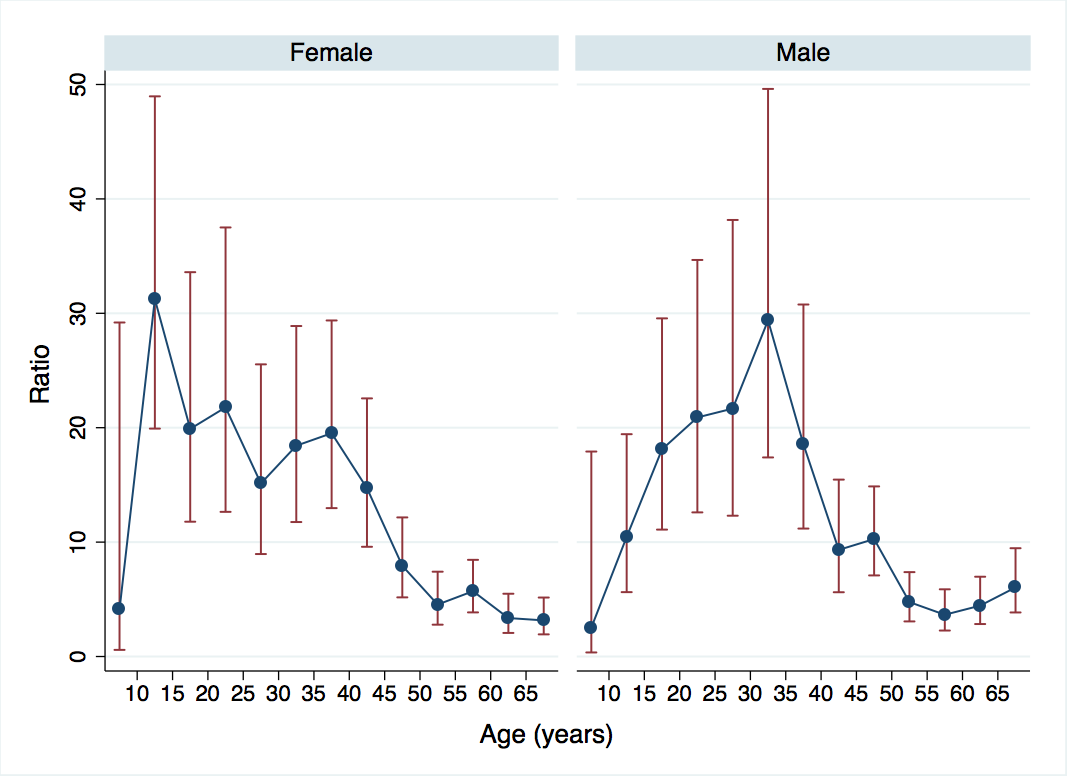

Supplement: S2 Fig — Plotted at mid-point of age group with 95% CIs. (TIF) [file pntd.0004033.s010.tif]

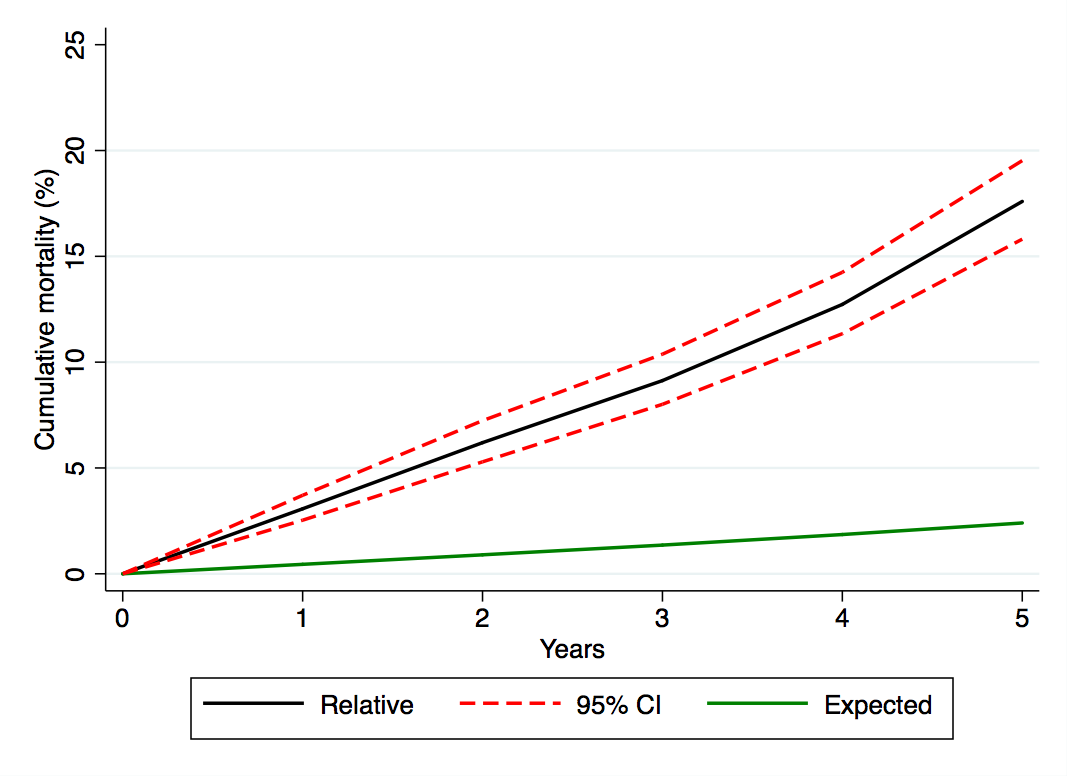

Supplement: S3 Fig — Plotted using local weighted scatter plot smoothing. (TIF) [file pntd.0004033.s011.tif]

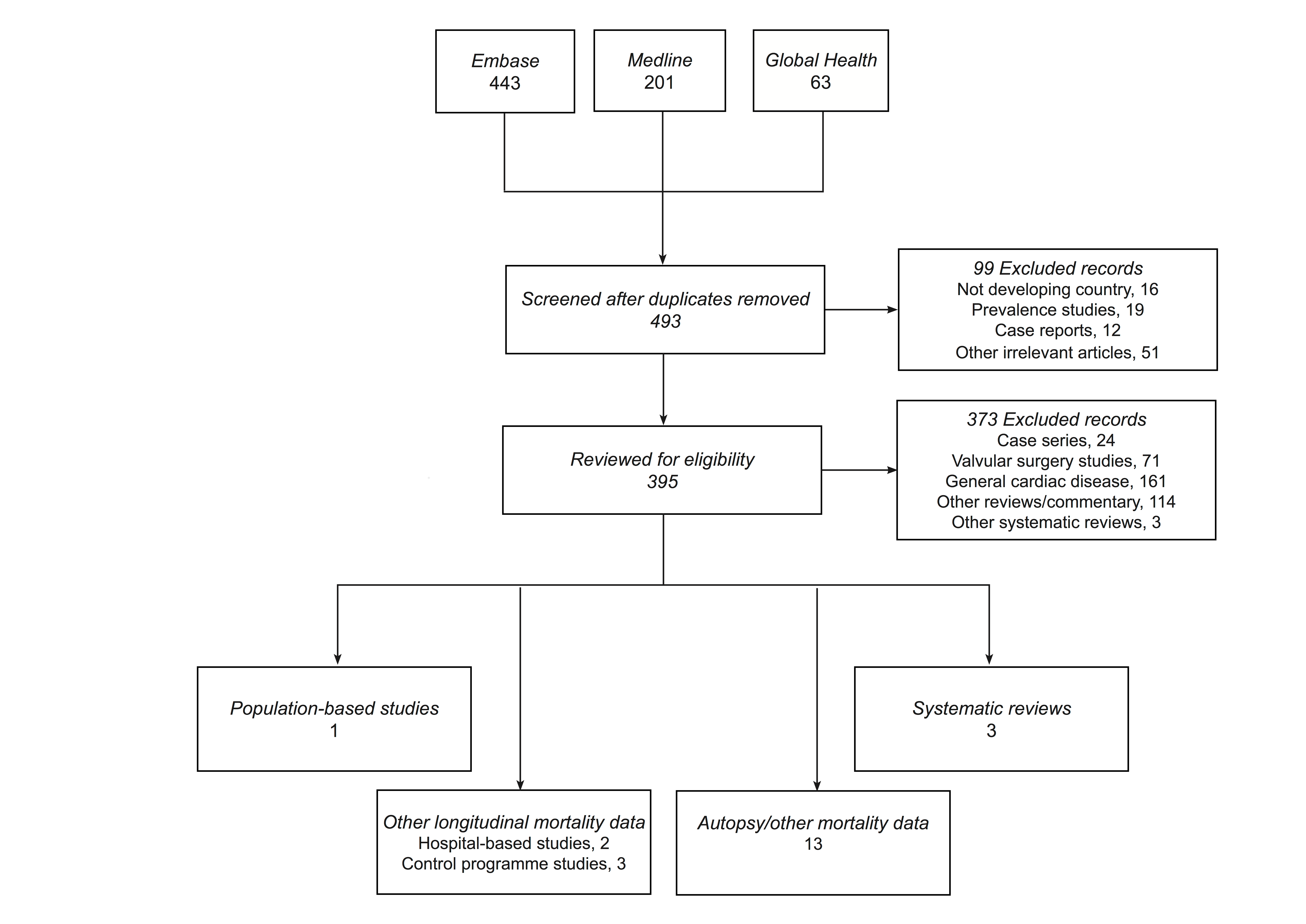

Supplement: S4 Fig — (TIF) [file pntd.0004033.s012.tif]
