# Supplementary material for: Rheumatic Heart Disease-Attributable Mortality at Ages 5–69 Years in Fiji: A Five-Year, National, Population-Based Record-Linkage Cohort Study
Source: PLoS Negl Trop Dis. 2015 Sep 15;9(9):e0004033. doi: 10.1371/journal.pntd.0004033 (PMC4570761; doi:10.1371/journal.pntd.0004033)
Supplement: S1 Box — (PDF) [file pntd.0004033.s013.pdf]

S1 Box. Calibration study for record linkage procedure

Although rarely used in practice, the patient information system has a merger tool that was introduced to allow clerical staff to join records referring to the same individual in an effort to address the problem of patients with multiple records. There were 1,406 records merged with at least one other record in the system referring to 1,332 individuals giving 1,262 duplicates, sixty-six triplicates and four quadruplicates.

Our record-linkage procedure was used tested for its ability to find these duplications using, as identifiers, the individual's name(s), father's name(s), year/date of birth, gender, locality of residence and date of death. None of these records had missing individual name(s), birth year or locality of residence, but 858 (61.0%) had missing father's name(s) and 97 (6.9%) had missing day and month of birth.

When preliminarily blocked by birth year and the opening six characters of the first or last names, 1,096,791 potential matches were found for 1,404 search records, and none were found for two. Of these potential matches, 9,338 pairs pertaining to 1,385 search records were shortlisted (Table). The procedure correctly identified 1,281 of 1,402 true mergers (sensitivity 91.4%, 95% CI 89.8-92.8%, Table), but identified an additional 871 false matches (specificity 99.92%, 95% CI 99.91-99.93%) giving a Receiver Operating Characteristic curve area 95.6% (95% CI 94.9-96.4%).

Table: **Contingency table for record-linkage status**

		Matched by linkage		
		Yes	No	Total
*Merged in PATIS**	Yes	1,281	121	1,402
	No	870	1,094,519	1,095,389
	Total	2,151	1,096,640	1,096,791

*Patient information system
