# Supplementary material for: Rheumatic Heart Disease-Attributable Mortality at Ages 5–69 Years in Fiji: A Five-Year, National, Population-Based Record-Linkage Cohort Study
Source: PLoS Negl Trop Dis. 2015 Sep 15;9(9):e0004033. doi: 10.1371/journal.pntd.0004033 (PMC4570761; doi:10.1371/journal.pntd.0004033)
Supplement: S2 Box — (PDF) [file pntd.0004033.s014.pdf]

S2 Box. Inclusion and exclusion criteria

Patients were eligible if:

1. They had been included in the control program register;
2. An echocardiogram of the patient had demonstrated findings consistent with RHD and/or a clinician had made an echocardiographic diagnosis;
3. They had been discharged from a hospital with the diagnosis;
4. They had died and the diagnosis was stated on the medical death certificate.

Patients were excluded if:

1. The diagnostic information in the control program register stated ”suspected” or ”borderline” (a term not equivalent in this context to its use in the World Heart Federation guidelines);
2. The only echocardiographic finding was an isolated morphological feature such as valve thickening, a non-specific finding;
3. The only diagnostic information dated from before their fifth birthday, before which time the disease is unusual;
4. They were registered as an overseas patient;
5. They had died before 1st January 2008.
